# Anti-Streptococcic Serum

**Published:** 1898-04-23

**Authors:** 


### ANTI-STREPTOCOCCIC SERUM.

The present position of our knowledge in regard to the therapeutic action of anti-streptococcic serum is well summed up by Dr. Louis Cobbett. As a result of laboratory research it has been shown (1) that anti-streptococcic serum possesses some power of protecting rabbits against the streptococcus used to immunise the animals which yielded the serum, but that there is reason to believe that this power may be easily lost. (2) That the serum has been shown to be incapable of protecting against every streptococcus which is pathogenic to man. (3) That much remains to be done before the serum can be recommended to be used in cases of human disease. We do not, as a rule, know, in any given case, whether the disease is due to the streptococcus or to some other organism, and even if we could make sure of this it would still be necessary to decide what kind of streptococcus it was due to, before the right serum could be applied. Until it has been shown beyond the possibility of doubt that the serum will protect animals from, and cure them of artificial streptococcal infections, it is not yet ready for the physician. It is, moreover, very important that we should discriminate between the position occupied by anti-diphtheritic serum, a remedy of surpassing value, and of that held by a host of other anti-serums for syphilis, tubercle, cancer, &c., whose value is as yet very uncertain.

